# Free‐living, continuous hypo‐hydration, and cardiovascular response to exercise in a heated environment

**DOI:** 10.14814/phy2.13672

**Published:** 2018-04-24

**Authors:** Kate S. Early, Conrad P. Earnest, Bailey Theall, Nathan P. Lemoine, Brian Harrell, Neil M. Johannsen

**Affiliations:** ^1^ School of Kinesiology Louisiana State University Baton Rouge Louisiana; ^2^ Department of Health, Physical Activity and Exercise Science Columbus State University Columbus Georgia; ^3^ Department of Health and Kinesiology Texas A&M College Station Texas; ^4^ Baton Rouge General Baton Rouge Louisiana

**Keywords:** Cardiovascular, heat stress, hydration, thermoregulation

## Abstract

Chronic dehydration (DEH) and heat stress combined with poor cardiovascular (CV) health may influence physiological responses to exercise. We examined the effects of free‐living induced hypo‐hydration on physiological responses to exercise in a heated environment and whether resting CV health is related to these changes. Participants (*N* = 16, 20.6 ± 1.2 years) were randomized to 3 days of voluntary fluid restriction (DEH) or intake (hydration [HYD]) followed by an exercise bout. CV health was assessed by flow‐mediated dilation (FMD), pulse wave analysis, and heart rate variability (HRV). HYD was assessed by weight, urine color, and specific gravity (USG). Exercise trials were conducted in a heated environment (30.3 ± 0.8°C, 27.4 ± 7.4% RH) on a cycle ergometer for 30 min. Heart rate (HR), weighted skin (*T*_sk_) and mean body temperature (*T*_b_) and skin blood flow (SBF) were assessed during exercise. Pre‐exercise weight (*P* < 0.005), urine color, and USG (*P* < 0.001) were different in between trials. HR was greater in DEH (153 ± 26 bpm) versus HYD (144 ± 23 bpm, *P* = 0.02) after exercise. No group differences were found, but a time interaction *P* < 0.001) for all temperature responses and time‐by‐trial interaction for *T*_re_ (*P* < 0.01) and *T*_sk_ (*P* < 0.001) was observed. Greater changes in *T*_re_ (*P* = 0.02) and *T*_sk_ (*P* < 0.01) were associated with increased FMD. Free‐living, continuous DEH alters weight, blood, and urine markers of HYD as well as HR response during exercise. Resting CV health was related to increased change in *T*_re_ and *T*_sk_, suggesting CV health plays a role in the mechanism of heat dissipation when DEH even in college‐age men and women.

## Introduction

Heat stress (heat‐related factors working on the body) and dehydration (DEH) present dual challenges to the cardiovascular (CV) system, hindering performance during exercise (Armstrong et al. 1985; Montain and Coyle 1992; Gonzalez‐Alonso et al. 1997). Reduced maximal aerobic capacity (*V*O_2max_) during exhaustive exercise in the heat may be characterized by increased heat storage, core temperature, and demand for adequate sweat production (Sawka et al. 1985; Crandall and Gonzalez‐Alonso 2010). In addition, previous research suggests that exercise in the heat when hypohydrated reduces cardiac output and blood pressure (BP), requiring a higher heart rate (HR) to compensate for losses in blood volume from sweat production (Gonzalez‐Alonso et al. 1997). Chronic DEH (persistent water loss resulting in ≥1% reduction in body weight) itself is associated with increased risk for CV disease (Chan et al. 2002; Sontrop et al. 2013), potentially due to increased oxidative stress from activation of the renin‐angiotensin aldosterone system (Fanelli and Zatz 2011), as well as sympathetic nervous system activation (Carter et al. 2005). Furthermore, thermoregulatory responses to heat stress (i.e., increased heat storage, decreased forearm blood flow) may be altered in individuals with chronic health conditions including type 2 diabetes (Kenny et al. 2013), obesity (Vroman et al. 1983), and heart failure (Balmain et al. 2016). Thus, chronic hypo‐hydration and heat strain (how body responds to heat stress) may have concomitant consequences on CV homeostasis and thermoregulation, which may be exacerbated by declining CV health.

Both the vascular endothelium and autonomic nervous system are involved in CV homeostasis. Noninvasive prognostic measurements of endothelial and autonomic function including flow‐mediated dilation (FMD) (Arnaoutis et al. 2017), pulse wave velocity (PWV) (Caldwell et al. 2017) and heart rate variability (HRV) (Carter et al. 2005; Castro‐Sepulveda et al. 2014) are decreased in response to DEH as well in CV disease. Vascular dysfunction arising from CV disease development may occur when hypohydrated partly due to increased release of angiotensin II and decreased nitric oxide bioavailability causing vasoconstriction (Dijkhorst‐Oei et al. 1999). Hypo‐hydration alone increases CV strain and is associated with decreased parasympathetic activity (Crandall et al. 2000). Increased catecholamines during exercise and heat stress may also contribute to increased sympathetic activation during exercise when hypo‐hydrated, thus exerting a presser effect in the vasculature (Gonzalez‐Alonso et al. 1995; Carter et al. 2005). However, the few studies examining CV health (measured by FMD, PWV, HRV) in relationship with hypo‐hydration and/or heat stress‐induced DEH through pharmacological or exercise interventions (Armstrong et al. 1985; Cheung and McLellan 1998; Arnaoutis et al. 2017).

Evidence suggests individuals only replace ~70% of total water lost during exercise in the heat (Greenleaf and Sargent 1965) and do not ingest sufficient fluid during physical activity to replace water losses and return to a euhydrated state. Therefore, individuals subject to heat strain may persistently dehydrate day to day, causing impairments in CV function measured by increased HR and BP (Gonzalez‐Alonso et al. 1997; Crandall and Gonzalez‐Alonso 2010). Few studies have examined the effects of free‐living chronic DEH due to persistent under‐consumption of fluids in relationship with their current CV health status (Armstrong 2012). The Adventist Health study suggested chronic hypo‐hydration was associated with increased risk of fatal coronary heart disease potentially due to increased blood viscosity, a known risk factor of CV disease (Chan et al. 2002). In addition, NHANES data has demonstrated high intake of water was inversely related to chronic kidney disease, suggesting euhydration is important to health (Sontrop et al. 2013). However, these studies used cross‐sectional data in an adult population with known CV disease risk factors already present.

The purpose of this study was to determine whether free‐living continuous hypo‐hydration, achieved by self‐determined fluid restriction over a 3‐day period, altered CV function (HR and BP), sweat and thermoregulatory (skin, rectal, and mean body temperature) responses during exercise in a heated environment. In addition, this study aimed to determine if resting CV health (FMD, PWV, HRV) was related to skin, rectal and mean body temperature, skin blood flow (SBF), and local sweat rate (LSR) during exercise. We hypothesized that CV function (HR and BP) and LSR would be blunted in a hypo‐hydrated state accompanying exercise in a heated environment due to increased CV strain and heat stress during exercise. Lastly, we hypothesized resting CV health evaluated by FMD, PWV, and HRV to be associated with CV function and thermoregulatory responses during exercise.

## Methods

### Participants

Sixteen participants (6 males, 10 females) aged 20.6 ± 1.2 years participated in this study. High‐risk individuals as categorized by the American College of Sports Medicine (ACSM), including those with known CV, pulmonary or metabolic disease, or signs/symptoms suggestive of disease were excluded to avoid adverse events during exercise (Pescatello and A. C. O. S. M. 2014). In addition, participants taking medications that might potentially influence fluid balance or CV function were also excluded. This study was approved by the Louisiana State's University's Institutional Review Board. All participants provided written informed consent prior to any study assessments.

### Experimental protocol

**Figure 1:**
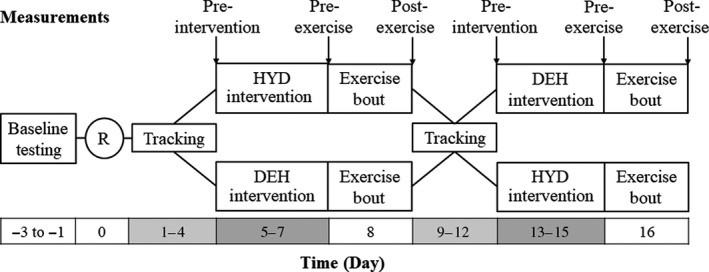
Crossover study design and randomization scheme following baseline testing. Participants were randomly assigned to dehydration (DEH) or hydration (HYD) protocols. Measurements were taken at pre‐intervention, pre‐exercise and post‐exercise.

A counterbalanced, cross‐over design was used whereby participants were randomized to two intervention periods and a total of 5 laboratory visits (Fig. 1). Experiments were conducted November through February, so subjects were not assumed to be heat‐acclimated. Participants were randomized to the hydration (HYD) or DEH trials after baseline testing. Participants spent 4 days tracking their perceived urine color and thirst from scales provided, and journaling normal, voluntary behaviors of food and fluid intake to determine their free living HYD standard (Armstrong et al. 1994). The purpose of the tracking period was to establish typical HYD status that could be replicated before the start of each intervention period and provide an adequate washout period of 4 days prior to starting the second intervention period. The randomly assigned intervention period included 3 days of either voluntary HYD or DEH where participants were encouraged to drink or restrict fluids to achieve each state, respectively. Participants continued to journal through this period to ensure each HYD state was maintained throughout the 3 days as opposed to 1 day prior to the exercise visit. To avoid interfering with free‐living behaviors participants received daily reminders to achieve their HYD status, but did not weigh in or provide urine samples. Exercise testing on the fourth intervention day was standardized to the same time of day to reduce effects of diurnal variation. To reduce the impact of menstrual cycle on HYD status and fluctuations in body temperature, female participants performed laboratory visits in the follicular phase of their menstrual cycle.

### Baseline testing

Participants arrived between 8:00 and 10:00 am for the baseline testing after an overnight fast of ≥10 h. In addition, participants were asked to avoid alcohol and exercise for ≥48 h prior to the baseline visit. Anthropometric measures included height, weight, and estimation of body surface area (BSA) using standard equations (Du Bois and Du Bois 1916). Body weight was used to determine changes incurred by changing HYD status during the intervention and exercise bout. Measurements of CV health (FMD, PWV, HRV) were performed in a quiet, dimly lit temperature controlled room. Conduit vessel function was assessed by FMD following current guidelines (Thijssen et al. 2011). With participants in the supine position and a three‐lead ECG placed on the wrists and leg to monitor cardiac cycle, FMD was induced by 5 min of forearm occlusion by inflation of a pneumatic cuff (Hokanson) to 200 mmHg, positioned ~1 cm distal to the olecranon process. Using ultrasonography (Logiq e Ultrasound, GE Healthcare, WI), the brachial artery was imaged for 30 sec prior to cuff release and for 3 min following return to normal, unrestricted blood flow. FMD was calculated as the percent change in brachial artery diameter. All ultrasound images were stored for subsequent analysis (ImageJ software, National Institutes of Health). BPs (systolic, diastolic, mean and pulse pressure) and systemic arterial stiffness (augmentation pressure and index) were measured with applanation tonometry (SphygomoCor, Version 8.0, AtCor Medical). Augmentation index (Aix) was calculated by the difference in the second systolic peak and diastolic pressure divided by the difference between the first systolic peak and diastolic pressure (×100%) (Wilkinson et al. 1998). HRV was examined according to the Task Force for Pacing and Electrophysiology (1996). Briefly, a HR monitor (Zephyr Technology Corp., Annapolis, MD) was worn during the resting and exercise visits to capture R‐R intervals and was analyzed using Kubios software 2.0 (Biosignal Analysis and Medical Imaging Group, Kuopio, Finland). Measurements of HRV were assessed from the last 5 min of the resting period in the supine position. Frequency components of HRV included log transformed low frequency (LF_ln_) and high frequency (HF_ln_) power, and normalized units (nu) LF and HF, calculated by dividing the LF and HF by the total spectral power minus the very low frequency, and low to high frequency ratio (LF/HF). HF is representative of parasympathetic activity while LF reflects both parasympathetic and sympathetic activity. Time domain measures included mean RR intervals, standard deviation of the RR intervals (SDNN) and the square root of the mean squared difference of RRs (RMSSD). SDNN is reflective of overall variability and RMSSD is an estimate of parasympathetic activity.

### Intervention protocol

During the intervention periods, participants were encouraged not to exercise strenuously and to avoid alcohol and prolonged outdoor activities. However, given the effect of caffeine withdrawal on CV parameters (Irwin et al. 2011), and the lack of data to support that caffeine promotes DEH (Paluska 2003), typical intake of caffeinated beverages was allowed. During the HYD trial participants were asked to drink adequate fluids prior to the exercise test in order to promote proper HYD indicated by perceived urine color (<3 out of 8) (Armstrong et al. 1998) and thirst (<3 out of 10). The U.S. military applies this method to estimate HYD status, which has been extensively researched in active military personnel (Armstrong et al. 1994). The thirst scale measures how thirsty participants felt ranging from 1 (not thirsty at all) to 10 (very, very thirsty). The urine color scale ranges from pale/clear urine (1) to dark orange/brown urine (8). During the DEH trial, participants were asked to dehydrate by restricting fluid intake to sustain a given thirst level (perceived thirst of 7 out of 10) and urine color (>4 out of 8) (Armstrong et al. 1998). If a participant's thirst exceeded a rating of seven they were instructed to drink just enough fluids to alleviate the sensation of thirst. In addition, participant's fluid intake during meals was restricted to 1 cup (250 mL) of fluid and complete fluid restriction the morning of the exercise test.

### Exercise testing protocol

Each exercise test consisted of a 30‐min steady‐state bout of exercise on a cycle ergometer (Monarch Ergomedice 828E) in a heated environmental chamber (30.3 ± 0.8°C, 27.4 ± 7.4% RH) with minimal convective airflow. Respiratory gases were monitored during exercise sessions using an integrated oxygen/carbon dioxide analyzer calibrated with standard gas mixtures (TruOne 2400, ParvoMedics, Inc., Sandy, UT). During the steady‐state exercise bout, pedal rate on the cycle ergometer was set to 60 rpm and a resistance factor necessary to elicit an individualized estimated work rate of 35.0 ± 0.9 W/m^2^ (62.7 ± 5.2 W) using ACSM cycle equations (Pescatello and A. C. O. S. M. 2014). No fluids were permitted during the exercise tests, and BP was measured at 10‐min intervals while a HR monitor was worn throughout the visit. During the exercise test, rectal temperature (*T*_re_), skin temperatures (*T*_sk_) and SBF were continuously monitored.

### Blood, urine and sampling

A 5 mL venous blood sample was drawn into lithium‐heparin tubes at baseline, pre‐intervention and pre‐ and post‐exercise. Whole blood was immediately analyzed for contents of hemoglobin spectrophotometrically using the cyanmethemoglobin method (Sigma‐Aldrich, MO) and hematocrit using the microcapillary technique in triplicate. Thereafter, blood was centrifuged and analyzed in triplicates for plasma electrolytes, including sodium (Na^+^), potassium (K^+^), and chloride (Cl^−^) (EasyLyte, Medica Corp., MA), and plasma osmolality (P_osm_) using vapor pressure osmometry (Wescor Elitech Group, Logan, Utah). Urine samples were analyzed for color, urine specific gravity (USG) (hand refractometer, NSG Precision Cells, Inc., Farmingdale, NY), urine electrolytes (EasyLyte), and osmolality (U_osm_) (Wescor). Perceived thirst and urine color were recorded on journals using a thirst scale (1 not thirsty at all, 10 very, very thirsty) and a urine color scale (Armstrong et al. 1994). During exercise, LSR of the upper back were measured during exercise with the technical absorbent technique (Morris et al. 2013). LSR is reported as the difference in pre‐ and postpatch weight, divided by the surface area (cm^2^) and duration of application (30 min) (mg/cm^2^ × min) (Morris et al. 2013). Sweat collected from the electrolyte‐free absorbent patch was centrifuged and analyzed for electrolyte concentrations using ion selective electrodes (EasyLyte, Medica Corp.).

### Skin temperature and blood flow

Skin thermometers (TSD202A, Biopac Systems, Santa Barbara, CA) sampled at 4 Hz were placed on four sites: the mid‐thigh, chest, mid‐biceps, and calf (Mitchell and Wyndham 1969). A rectal thermometer was inserted 10 cm to continuously record rectal temperature (*T*_re_) (TSD202F, Biopac Systems). Weighted skin temperature (*T*_sk_) was estimated using the equation of Ramanathan (1964). Mean body temperature (*T*_b_) was calculated from rectal (*T*_re_) and *T*_sk_ using the equation *T*_b_ = 0.8T_re_ + 0.2*T*_sk_ (Colin et al. 1971). The change in temperatures was calculated by the difference in skin temperatures at that start of exercise (*T*_0_) and maximal *T*_sk_ (*T*_max_). SBF was measured real‐time (20 Hz) during exercise using a single‐point laser Doppler flowmeter (Perimed, Stockholm, Sweden). The laser Doppler probe was affixed to the flexor aspects of the forearm (muscle belly of the brachioradialis) using adhesive and surgical tape. SBF slope was determined by the difference between perfusion units at the onset of SBF rise and the plateau of perfusion units, divided by the time.

### Statistical analysis

Statistics were performed in JMP statistical software 12 (SAS Institute Inc., Cary, NC). Independent Student *t*‐tests were performed to describe sex differences in participant characteristics and CV measures. One‐way and two‐way (trial‐by‐time) repeated measures analysis of variance were performed to determine differences between HYD and DEH and across the tracking, intervention, and exercise time points. Significant main or interaction effects were further evaluated using Bonferroni corrected post hoc testing where appropriate. Pearson correlations were used to examine the relationships between the thermoregulatory (*T*_re_, *T*_sk_, *T*_b_ SBF) and CV (HRV, PWV, FMD) variables. Data are displayed as mean ± standard deviation (SD) or mean changes and 95% confidence interval as appropriate. Significance was established at *P* < 0.05.

## Results

### Participants

Participants (*n* = 16, 20.6 ± 1.2 years) were of normal body weight (67.9 ± 10.9 kg), body mass index (23.8 ± 3.6 kg/m^2^) and had a normal systolic (109 ± 9 mmHg) and diastolic (72 ± 7 mmHg) BP. Males had a larger BSA (1.92 ± 0.08 vs. 1.69 ± 0.12 m^2^) than females (*P* < 0.05). Resting CV function was assessed by FMD, PWV, and HRV and is presented in Table 1. Resting (*P* = 0.001) and peak (*P* = 0.01) artery diameter was greater in males compared to females, but no difference was observed in FMD (*P* = 0.30) between sexes.

**Table 1:** Screening cardiovascular measures

	Male (*n* = 6)	Female (*n* = 10)	All (*n* = 16)	*P*‐value
HR variability				
Resting HR (bpm)	62 ± 11	64 ± 6	63 ± 8	0.68
Mean RR (msec)	1022 ± 188	952 ± 100	978 ± 138	0.34
SDNN (msec)	128 ± 70	89 ± 41	103 ± 55	0.18
RMSSD (msec)	100 ± 53	84 ± 49	90 ± 50	0.55
LF_ln_	8.2 ± 0.8	7.4 ± 0.9	7.7 ± 0.9	0.13
HF_ln_	8.1 ± 1.2	7.5 ± 1.3	7.7 ± 1.3	0.36
LF_nu_	51.9 ± 16.2	48.6 ± 13.9	49.8 ± 14.3	0.68
HF_nu_	48.2 ± 16.2	51.3 ± 13.9	50.1 ± 14.4	0.69
LF/HF	1.32 ± 0.95	1.09 ± 0.61	1.17 ± 0.73	0.56
Vascular measures				
Aortic SBP (mmHg)	95 ± 9	90 ± 5	92 ± 7	0.13
Aortic DBP (mmHg)	74 ± 8	69 ± 5	71 ± 7	0.18
MAP (mmHg)	84 ± 8	79 ± 5	80 ± 6	0.11
AP (mmHg)	−1.6 ± 4.5	0.6 ± 3.2	−0.3 ± 3.7	0.29
AIx (%)	−3.5 ± 6.3	4.6 ± 12.4	1.5 ± 11.7	0.19
PWV (m/sec)	6.8 ± 0.9	6.4 ± 1.0	6.6 ± 0.9	0.43
Artery diameter (mm)	4.25 ± 0.55	3.26 ± 0.42^a^	3.63 ± 0.67	0.001
Peak artery diameter (mm)	4.49 ± 0.61	3.43 ± 0.47^a^	3.79 ± 0.71	0.01
Absolute change (mm)	0.25 ± 0.09	0.23 ± 0.07	0.24 ± 0.08	0.74
BAFMD (%)	5.68 ± 1.66	6.71 ± 2.00	6.67 ± 2.33	0.30

Mean ± standard deviation (range). BAFMD, brachial artery flow mediated dilation; HR, heart rate; RR, R‐to‐R interval; SDNN, standard deviation of RR intervals; LF_nu_, low frequency normalized units; HF_nu_, high frequency normalized units; LF/HF, low‐to‐high frequency ratio; SBP, systolic blood pressure; DBP, diastolic blood pressure; AP, augmentation pressure; AIx, augmentation index; PWV, pulse wave velocity.

a Significant difference between genders (*P* < 0.05).

### Tracking and intervention periods

Perceived urine color (*P* = 0.89), perceived thirst (*P* = 0.14), weight (*P* = 0.87), USG (*P* = 0.50), and P_osm_ (*P* = 0.13) were not different between tracking periods. Participants recorded perceived urine color across each day of represented was different between HYD statuses. Day 1 perceived urine color when comparing HYD to DEH was 3 ± 1 versus 4 ± 1 (*P* = 0.04), day 2 was 2 ± 1 versus 4 ± 1 (*P* = 0.002), and day 3 was 2 ± 1 versus 6 ± 1 (*P* < 0.001). From the start of the intervention period (pre‐intervention) to the start of exercise (pre‐exercise) there was a 0.1 ± 0.7% change in body weight during HYD intervention compared to −0.7 ± 0.9% in the DEH intervention. The DEH intervention produced a lower body weight, higher USG and P_osm_ (*P* < 0.05) compared to the HYD intervention (Table 2).

**Table 2:** Pre‐intervention and pre‐ and post‐exercise indices of hydration (*n* = 16)

	Pre‐intervention		Pre‐exercise		Post‐exercise	
	HYD	DEH	HYD	DEH	HYD	DEH
Weight (kg)	67.7 ± 11.1	67.9 ± 11.0	67.9 ± 10.5	67.2 ± 10.8^a^, ^b^	68.7 ± 10.6	66.1 ± 10.8^a^^,^ c
USG	1.022 ± 0.004	1.020 ± 0.007	1.016 ± 0.008^a^	1.023 ± 0.007^b^	1.015 ± 0.009^a^	1.024 ± 0.007^a^^,^ c
Urine color	3 ± 1	4 ± 1	3 ± 1	5 ± 2^a^, ^c^	3 ± 1	4 ± 1^c^
U_osm_ (mOsm/kg)	648 ± 362	513 ± 414	210 ± 239^a^	223 ± 273^a^	204 ± 240^a^	247 ± 341^a^
P_osm_ (mOsm/kg)	290 ± 11.6	294 ± 12.2	282 ± 11^a^	301 ± 8^a^, ^b^	292 ± 10^a^	298 ± 9
Hematocrit (%)	47 ± 5	47 ± 4	49 ± 8	46 ± 4	49 ± 7	46 ± 3^c^

Mean ± standard deviation. HYD, hydration; DEH, dehydration; USG, urine specific gravity; U_osm_, urine osmolality; P_osm_, plasma osmolality.

a Different form pre‐intervention *P* < 0.05.

b Different between pre‐exercise trials (*P* < 0.05).

c Different between post‐exercise trials (*P* < 0.05).

### Exercise sessions

The average workload of participants during the exercise trials was 35.0 ± 0.9 W/m^2^ (62.7 ± 5.2 W). Total work (401 ± 57 vs. 390 ± 46 kJ/m^2^, *P* = 0.45) performed was note different between HYD and DEH exercise trials. Oxygen consumption (HYD vs. DEH: *V*O_2_ 1.16 ± 0.29 vs. 1.16 ± 0.15 L/min; *P* = 0.92) and respiratory exchange ratio (RER) (HYD vs. DEH: RER 0.88 ± 0.03 vs. 0.87 ± 0.04; *P* = 0.55) were similar between exercise trials. Ambient temperature (HYD vs. DEH, 30.6 ± 0.8 vs. 30.4 ± 0.9°C, *P* = 0.46) and relative humidity (HYD vs. DEH, 28.3 ± 7.3% vs. 26.5 ± 7.6%, *P* = 0.48) were also not different between exercise trials. HR after 10 min of exercise was not different between trials (*P* = 0.19), but after 20‐min (150 ± 27 vs. 142 ± 23 bpm, *P* = 0.02) and at 30 min (153 ± 26 vs. 144 ± 23 bpm, *P* = 0.02) HR was greater in the DEH trial compared to the HYD trial. Similarly, systolic BP response tended to be greater after 20 and 30 min of exercise in DEH compared to HYD (20‐min: 143 ± 10 vs. 137 ± 5 mmHg, *P* = 0.17; 30 min 148 ± 11 vs. 137 ± 10 mmHg, *P* = 0.15). Diastolic blood pressure was not different between DEH and HYD after 20‐min or 30‐min of exercise (20‐min: 83 ± 6 vs. 83 ± 6 mmHg, *P* = 0.89; 30‐min: 85 ± 5 vs. 83 ± 3 mmHg, *P* = 0.51).

Complete *T*_re_, *T*_sk_, *T*_b_, and SBF data were determined in 12 participants (5 males, 7 females), and sweat samples were assessed in 10 participants (5 males, 5 females); 4 participants did not collect data on all skin temperature sites due to detached thermistors while 6 participants did not produce enough sweat to measure LSR or electrolytes. LSR (HYD vs. DEH: 4.6 ± 3.5 vs. 4.3 ± 4.2 g/cm^2^ per min, *P* = 0.78) and sweat [Na^+^] (HYD vs. DEH: 56.3 ± 28.7 vs. 59.9 ± 26.1 mmol/L, *P* = 0.76) were not different between exercise trials.

**Figure 2:**
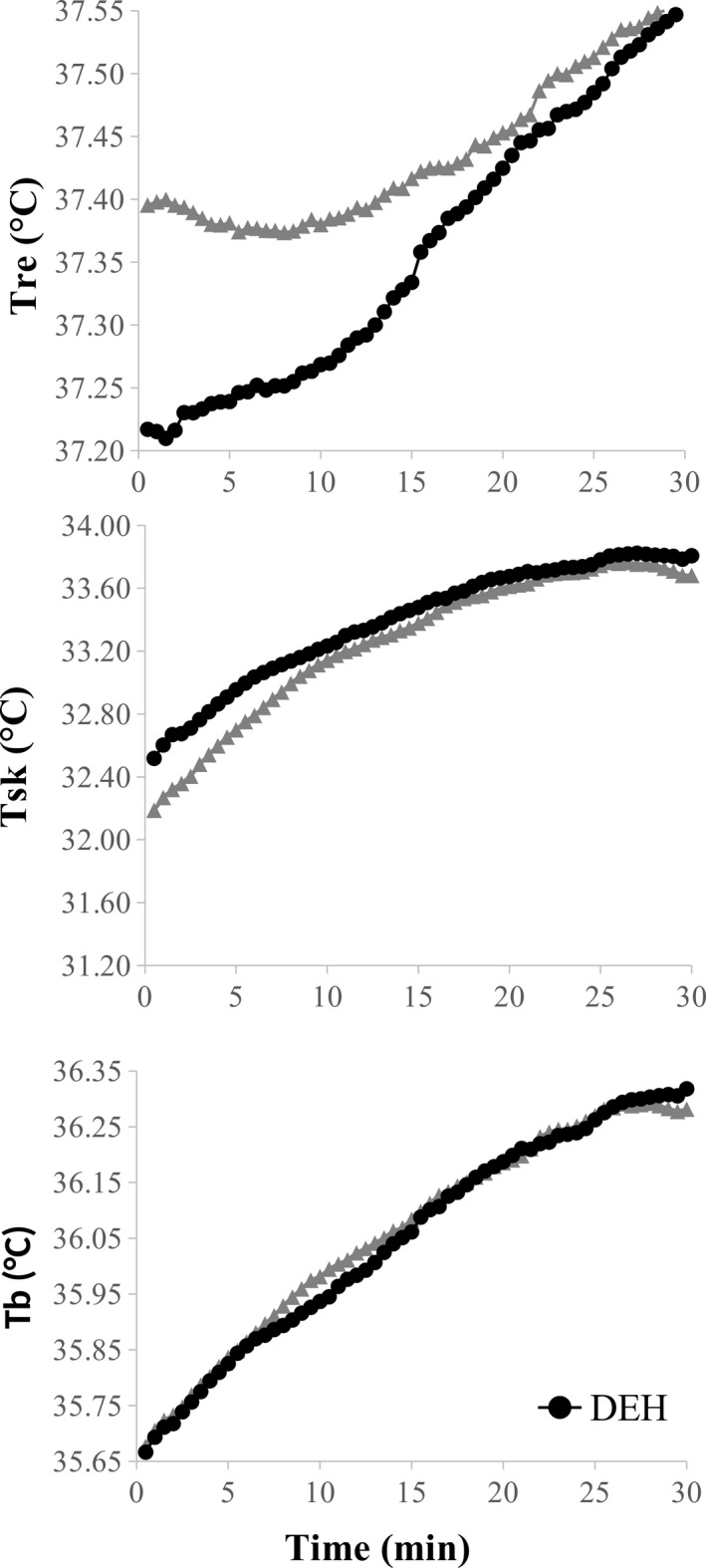
Mean temperature changes (°C) for a 30‐min exercise bout. *T*_re_ rectal temperature, *T*_sk_ weighted skin temperature, and *T*_b_ total body temperature. Main effect time for all temperatures (*P* < 0.001) and *T*_re_ (*P* < 0.001) and *T*_sk_ (*P* < 0.01) time and trial interaction.

The mean temperature changes across time are presented in Figure 2. At the start of exercise, *T*_re_ (37.32 ± 0.17 vs. 37.19 ± 0.34°C, *P* = 0.07), *T*_sk_ (32.38 ± 0.67 vs. 32.41 ± 0.55°C, *P* = 0.50), and *T*_b_ (35.73 ± 0.2 vs. 35.61 ± 0.37°C, *P* = 0.33) were not different between HYD and DEH trials. The main effect of time was significant for *T*_re_, *T*_sk_, and *T*_b_ (*P* < 0.001). There was a significant time by trial interaction between for *T*_sk_ (*P* < 0.01) and *T*_re_ (*P* < 0.001), but not for *T*_b_ (*P* = 0.90). SBF response lower in DEH compared to HYD (2.6 ± 1.2 vs. 3.7 ± 2.9 PU, *P* = 0.24).

### CV health relationships to physiological variables

**Figure 3:**
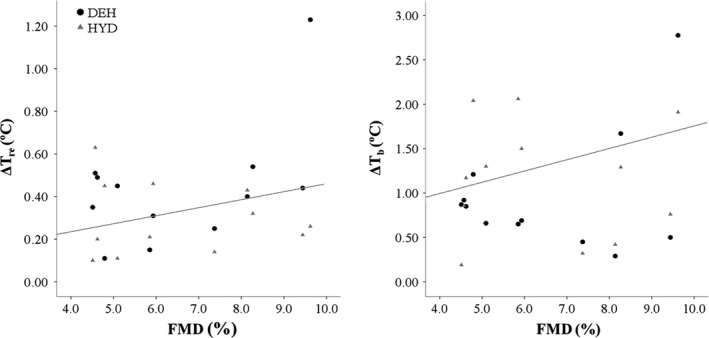
Relationship of brachial artery flow mediated dilation (FMD) to change in rectal (*T*_re_) and mean body (*T*_b_) after a 30‐min exercise bout. Significant correlation was found between FMD and *T*_re_ (*P* = 0.04) and *T*_b_ (*P* < 0.01).

FMD was significantly associated with Δ*T*_re_ (*r* = 0.41, *P* = 0.04) and Δ*T*_b_ (*r* = 0.54, *P* < 0.01). Those with a greater FMD had a greater change in *T*_re_ and *T*_b_ during exercise (Fig. 3). AIx was significantly associated with SBF (*r* = −0.40, *P* = 0.04) suggesting those with a high AIx had a smaller change in SBF during exercise. No other relationships between CV health (FMD, PWV, HRV) and thermoregulatory (*T*_re_, *T*_sk_, and *T*_b_) and CV (SBF) responses were observed.

## Discussion

This study sought to determine whether free‐living, chronic DEH, achieved by fluid restriction as opposed to acute exercise or pharmacological intervention, would alter CV function during exercise in a heated environment. Secondly, this study questioned whether CV health, measured by FMD, PWV, and HRV was related to the CV and thermoregulatory responses during exercise in the heat. The findings this study concluded that 3 days of self‐determined chronic, persistent DEH intervention alters weight, urine and blood markers of HYD and impaired CV response (HR and BP) during exercise in a heated environment. There was a significant main of effect of time on temperature markers (*T*_re_, *T*_sk_, *T*_b_) and a significant interaction in *T*_re_ and *T*_sk_ between trials after exercise, suggesting that when in a hypo‐hydrated state the body may alter thermoregulation differently during exercise compared to a hydrated state. Participants had a lower absolute change in diameter and FMD compared to previous research (Juonala et al. 2008). PWV was not different between sexes (*P* = 0.43), and was comparable to previous research (Reference Values for Arterial Stiffness C 2010). The time domain measurements SDNN and RMSSD and the frequency domain measures LF_ln_ and HF_ln_ were higher compared to normative data (Nunan et al. 2010). Although this population was overtly healthy, FMD and HRV tended to in the lower ranges of normative data of previous research, suggesting changes in CV health may already be beginning in this population. In addition, greater FMD was related to a greater change in *T*_re_ and *T*_sk_ during exercise in the heat, suggesting CV health plays a role in the rate in which body temperature increases during exercise. Together these results suggest that chronic, persistent DEH can produce alterations in HYD markers and CV and thermoregulatory responses to exercise in the heat might be influenced by CV health.

Through the tracking periods, participants maintained the same HYD status as measured by weight, perceived thirst and urine color, and USG at the pre‐intervention visits, suggesting participants started HYD interventions in similar states (Table 2). USG for HYD and DEH were similar to the work of (Perrier et al. 2013) where free‐living high drinkers and low drinkers were observed. Furthermore, the similar states at the pre‐intervention visits suggest that the second randomized tracking period was sufficient time to return to baseline HYD status.

According to current recommendations, the combination of weight, urine color, and thirst provide a strong indication of hypo‐hydration (Shirreffs 2003; Pescatello and A. C. O. S. M. 2014). During the HYD and DEH interventions, participants achieved a small change in body weight (HYD 0.1 ± 0.7% vs. DEH −0.7 ± 0.9%) through free‐living, self‐determined fluid intake. The change in body weight over the course of the self‐determined HYD intervention produced similar results to previous free‐living populations in a similar time frame (Armstrong et al. 2010). In comparison, the majority of studies involving DEH achieve a decrease in 2–3% in body weight acutely (Montain and Coyle 1992; Armstrong et al. 1997) using exercise to promote DEH. Despite these small chronic changes in HYD status, weight, urine color, and perceived thirst were different between pre‐HYD and pre‐DEH exercise trials, suggesting participants achieved euhydrated and hypo‐hydrated states during the intervention naturally and in as few as 3 days. In addition, plasma osmolality and USG were also different between pre‐HYD and pre‐DEH exercise trials, also suggesting participants were indeed hypo‐hydrated. In terms of real world application individuals who chronically consume less fluids day to day may fail to compensate for fluid losses, and may develop physiological mechanisms such as increased circulating stress hormones to compensate for mild chronic hypo‐hydration (Perrier et al. 2013).

The exercise bouts for both HYD and DEH trials were performed in similar warm and humid environments. The exercise environment was comparable to previous studies (Montain and Coyle 1992; Armstrong et al. 1997; Kenefick et al. 2010), but was less heat stress than several existing studies that induced DEH through exercise (Vroman et al. 1983; Gonzalez‐Alonso et al. 1997). When exercise was performed in the hypo‐hydrated state, as anticipated HR was significantly higher (*P* = 0.02), suggesting a hypo‐hydrated state is more taxing on the CV system compared to a euhydrated state (Montain and Coyle 1992; Gonzalez‐Alonso et al. 1997). Although not significant, SBF was lower during the DEH trial, which DEH and heat stress may independently and concomitantly reduce SBF and heat dissipation (Gonzalez‐Alonso 1998) and ultimately may impair aerobic performance (Kenefick et al. 2010). However, reductions in SBF do not necessarily result in impaired thermoregulation (Vroman et al. 1983; Cramer et al. 2017). Interestingly, an interaction was found between time and trial in *T*_re_ and *T*_sk_, suggesting thermoregulatory mechanisms that are prompted during exercise manage heat strain more efficiently when euhydrated compared to dehydrated. However, the environment and work rate may not have produced enough stress on the CV system to significantly alter SBF or sweat response. Previous studies demonstrate sweat rate varies widely depending on exercise intensity, environment and clothing worn (Tankersley et al. 1992; Gill et al. 2000). In addition, the participants’ baseline fitness level may have impacted their sweat rate. Increased aerobic fitness may increase the likelihood the individuals tolerate heat exposure by increasing sweating (Cheung and McLellan 1998).

The negative effects of hypo‐hydration on performance are well documented, yet little evidence relates CV health and hypo‐hydration during exercise. This study is demonstrates a link between vascular function and change in *T*_re_ and *T*_b_ response when exercising. Increased FMD was related to increased change *T*_sk_ and *T*_b_ during exercise, suggesting the conduit vasculature might have greater ability to direct blood flow to the exercising limbs and in turn skin. Although exact mechanisms have not yet been elucidated, proper HYD may ensure a greater blood volume, which would generate a greater shear stress on vasculature. In addition, a hydrated state might reduce sympathetic activation due to adequate blood flow and viscosity to the skin during exercise in the heat. Other researchers have demonstrated in diseased populations such as type II diabetes (Kenny et al. 2013) and obese patients (Vroman et al. 1983) an impaired thermoregulation during exercise in the heat and a hindered ability to dissipate heat. Although changes in CV health were minimal in this young, overtly healthy group, CV health might exacerbate the CV and thermoregulatory response to exercise in the heat. Furthermore investigation is required into the relationship of thermoregulation and CV health is warranted.

An increased AIx was associated with decreased SBF, suggesting increased arterial stiffness may alter the body's ability to direct blood flow to the skin during exercise. Passive heat stress representative of increasing core temperature seen during exercise has been found to reduce arterial stiffness to a greater extent in those with greater resting arterial stiffness (Ganio et al. 2011), suggesting CV health plays a role in thermoregulation. Although no relationship was observed in HRV and thermoregulation, participants had decreased parasympathetic activity compared to previous research (Nunan et al. 2010), suggesting greater autonomic imbalance at rest. In rats subjected to heat stress with and without DEH, dehydrated rats had increased LF, suggesting higher sympathetic activity when dehydrated (Matthew et al. 2004). Similarly, Crandall et al. (2000) found increased LF and reduced vagal tone when participants were subjected to whole body heat stress (Crandall et al. 2000). Despite thermoregulatory demands during exercise and heat stress, withdrawal of vagal tone may allow for contraction of SBF in order to redirect blood flow to the core, and increase the rate at which heat is stored (1993). These effects may be further exacerbated by DEH and impaired vascular health.

A limitation of this study was participant compliance and awareness with the interventional protocol. HYD and/or DEH was voluntary and did not include morning urine or weigh‐ins during the intervention, which may have limited the magnitude of change in body weight and HYD status. Yet, blood and urine markers of HYD status were influenced with the small changes produced without interfering with free‐living, daily habits. This study also delivered a low environmental stress and work rate and duration during the exercise bouts compared to current literature in individuals that may have prior heat acclimatization (Gonzalez‐Alonso et al. 1997; Carter et al. 2005; Cheuvront et al. 2005). In this study, greater heat strain and duration of exercise may have elicited greater differences in CV and thermoregulatory responses between HYD states. We tended to begin observing these differences at the end of the 30‐min exercise bouts, suggesting even under moderate heat strain and DEH CV changes are occurring. Lastly, the participants were overtly free from CV disease suggesting the relationship between DEH and CV health during exercise may have been less apparent. HYD status itself may be a confounding factor to measurements of CV health (Caldwell et al. 2017). Despite these limitations, this study used self‐determined fluid intake to induce changes in HYD status which is more indicative of free‐living conditions.

In conclusion, free‐living progressive, chronic DEH for 3 days produced changes in weight, urine and blood markers of HYD. CV (HR and BP) and thermoregulatory changes during exercise (*T*_re_ and *T*_sk_) were altered depending on HYD status. Improved CV function measured by FMD may be related to the ability to better deliver blood flow to the skin during exercise in the heat. Future research should continue to examine the impact of resting CV function or disease status on CV and thermoregulatory responses to exercise, particularly in a free‐living hypo‐hydrated state.

## Conflict of Interest

None declared.
